# Dose Effects of Orally Administered *Spirulina* Suspension on Colonic Microbiota in Healthy Mice

**DOI:** 10.3389/fcimb.2019.00243

**Published:** 2019-07-05

**Authors:** Jinlu Hu, Yaguang Li, Sepideh Pakpour, Sufang Wang, Zhenhong Pan, Junhong Liu, Qingxia Wei, Junjun She, Huaixing Cang, Rui Xue Zhang

**Affiliations:** ^1^School of Life Sciences, Northwestern Polytechnical University, Xi'an, China; ^2^Department of General Surgery, First Affiliated Hospital of Xi'an Jiaotong University, Xi'an, China; ^3^Faculty of Applied Science, University of British Columbia, Kelowna, BC, Canada; ^4^Institute of Medical Research, Northwestern Polytechnical University, Xi'an, China; ^5^Princess Margaret Cancer Center, University of Health Network, Toronto, ON, Canada

**Keywords:** oral delivery, microalgae, large intestine, prebiotics, microorganisms, prevention, 16s rDNA sequencing

## Abstract

Oral supplemented nutraceuticals derived from food sources are surmised to improve the human health through interaction with the gastrointestinal bacteria. However, the lack of fundamental quality control and authoritative consensus (e.g., formulation, route of administration, dose, and dosage regimen) of these non-medical yet bioactive compounds are one of the main practical issues resulting in inconsistent individual responsiveness and confounded clinical outcomes of consuming nutraceuticals. Herein, we studied the dose effects of widely used food supplement, microalgae *spirulina* (*Arthrospira platensis*), on the colonic microbiota and physiological responses in healthy male *Balb/c* mice. Based on the analysis of 16s rDNA sequencing, compared to the saline-treated group, oral administration of *spirulina* once daily for 24 consecutive days altered the diversity, structure, and composition of colonic microbial community at the genus level. More importantly, the abundance of microbial taxa was markedly differentiated at the low (1.5 g/kg) and high (3.0 g/kg) dose of *spirulina*, among which the relative abundance of *Clostridium XIVa, Desulfovibrio, Eubacterium, Barnesiella, Bacteroides*, and *Flavonifractor* were modulated at various degrees. Evaluation of serum biomarkers in mice at the end of *spirulina* intervention showed reduced the oxidative stress and the blood lipid levels and increased the level of appetite controlling hormone leptin in a dose-response manner, which exhibited the significant correlation with differentially abundant microbiota taxa in the cecum. These findings provide direct evidences of dose-related modulation of gut microbiota and physiological states by *spirulina*, engendering its future mechanistic investigation of *spirulina* as potential sources of prebiotics for beneficial health effects *via* the interaction with gut microbiota.

## Introduction

The gut microbiota is a complex and functional ecological community and plays an important role in influencing the physiological states, disease susceptibilities, and even therapeutic efficacies (Flint et al., 2012; Lozupone et al., 2012; Geller et al., 2017). The homeostasis of the gut microbial community in terms of its distribution, diversity, species composition and metabolic output contributes to the net benefits of host health (Flint et al., 2012; Sommer and Backhed, 2013). Yet, the gut microbiota is frequently shaped under both host and environmental selective pressures, such as host genotype, intestinal barrier (e.g., mucus layers, IgA, and epithelia-associated immune cells), colonic environments (e.g., intestinal pH and oxygen gradients and bile acids re-absorption), life styles and living conditions (e.g., smoking, geographical location, and surgery) of which diet exerts a large effect on the microbial colonization and its relative abundance (Spor et al., 2011; Wu et al., 2011; Thursby and Juge, 2017). Dysbiosis as a result of the disruption to the overall state of gut microbiota (e.g., antibiotics utilization) has been associated with the pathogenesis of many chronic diseases, such as cancer, inflammatory bowel disease (IBD), cardiovascular diseases, obesity, and diabetes (Guinane and Cotter, 2013; de Clercq et al., 2016; Tang et al., 2017).

To sustain or restore the intestinal bacterial homeostasis in healthy individuals or disease states, several approaches have been implemented, including the oral supplementation of probiotics, prebiotics and synbiotics (Verdu, 2009; Quigley, 2018), fecal microbiota transplantation (Zipursky et al., 2012; Li et al., 2016; Bilinski et al., 2017), bacterial consortium transplantation (Li et al., 2015) as well as bacteriocins and bacteriophage targeted antimicrobial therapies (Mills et al., 2017). Especially, orally supplementing non-medical nutraceuticals derived from the food sources have been widely used for disease prevention and amelioration of disease symptoms due to its potential capacity of promoting the growth of commensal gut microorganisms (Cencic and Chingwaru, 2010; Laparra and Sanz, 2010; Wang et al., 2019). Nevertheless, the meta-analysis of humans and animals studies reveals inconsistent individual responsiveness and clinical outcomes of consuming food supplements or dietary compounds (Gibson et al., 2017; Quigley, 2018). One of the main practical issues in the evaluation of nutraceuticals is lack of fundamental quality control and authoritative consensus (e.g., formulation, route of administration, dose, and dosage regimen) of these non-medical yet bioactive compounds. In addition, there is a large knowledge gap of cause-and-effect relationships between marketed nutraceutical products, the change of microbial population and the physiological benefits.

In the current study, we investigated *spirulina*, one of the most commonly consumed microalgae as food supplements worldwide (de Jesus Raposo et al., 2016), because of its potential benefits of nutritional values and therapeutic properties in human health (Khan et al., 2005; Nicoletti, 2016). Oral supplementation of *spirulina* in human studies have shown to potentiate the innate immune system, ameliorate hyperlipidemia, reduce the body mass, improve antioxidant status, and enhance anti-inflammatory and antihypertensive effects (Hirahashi et al., 2002; Khan et al., 2005; Lu et al., 2006; Torres-Duran et al., 2007; Mazokopakis et al., 2014; Ngo-Matip et al., 2014; Yogianti et al., 2014; Szulinska et al., 2017). Although the underlying mechanism of claimed biological functions of *spirulina* has not yet been fully understood yet, recent studies in healthy and disease animal models have shown that *spirulina* can modulate the composition of gut microbiota (e.g., *Lactaobacilli* and *Roseburia*) that may link to improved health status (Rasmussen et al., 2009; Yusuf et al., 2016; Neyrinck et al., 2017). However, despite of its widespread global use by the general populations, the quantitative characterization of dose-related effects of *spirulina* on the safety and effectiveness remains unknown.

**Figure 1:**
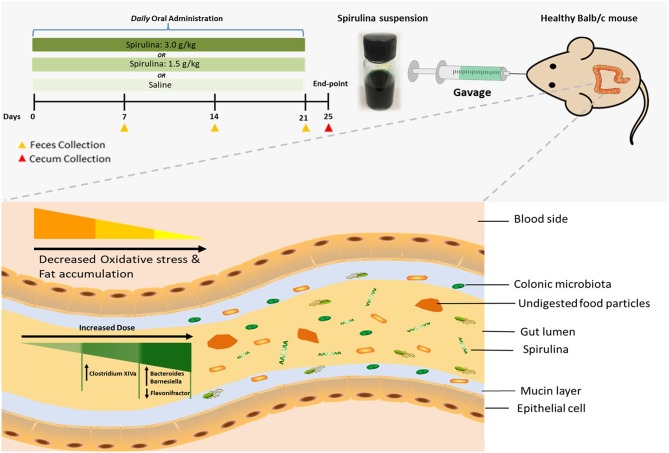
Schematic illustration of dose-dependent modulation of colonic microbiota and physiological responses in healthy mice *via* oral administration of *spirulina* suspension. **Top panel:** aqueous *spirulina* is orally administered to healthy male *Balb/c* mice at the low (1.5 g/kg) or high (3.0 g/kg) doses *daily* for consecutive 24 days. The change of colonic microbiota is identified from their fecal and cecal samples collected on day of 7th, 14th, 21st, and 25th post-treatment using high-throughput 16S rDNA sequencing. The status of oxidative stress and lipid profile are determined using various metabolic blood biomarkers (MDA, SOD, TG, TC, and leptin). **Bottom panel:** undigested components of *spirulina* (green color and spiral shape) are transited into the lumen of the distal large intestine where most of the gut microbial species colonize. Orally administered *spirulina* suspension alters the specific genus of colonic microbiota in a dose-dependent manner (triangle with gradient green color) in mice. At the low dose, strict anaerobes *Clostridium Cluster XIVa* is increased, whereas the high dose increases the abundance of *Bacteroides* and *Barnesiella* and decreases *Flavonifractor*. Oral intake of *spirulina* also effectively reduces the oxidative stress and fat accumulation (triangle with gradient yellow color) that is believed to relate to many chronic diseases, such as cancer, inflammatory bowel disease and cardiovascular disease.

Thus, to investigate whether the doses of *spirulina* alter on the gut microorganisms, which in turn affect physiological responses, orally administered *spirulina* at low and high doses in healthy mice were performed for a short period of intervention (Figure 1). The α- and β-diversity of colonic microbiota from fecal and cecal samples at designed times were evaluated by small subunit ribosomal DNA (16s rDNA) sequencing. Differentially abundant bacteria organisms were identified at the genus-level among different treatment groups using a quantitative computational method. Various health related indicators, including the body weight and biological markers, such as malondialdehyde (MDA), superoxide dismutase (SOD), total cholesterol (TC), total triglycerides (TG), and leptin, were also measured in the serum.

## Materials and Methods

### Animal Maintenance

All animal experiments were approved by the Institutional Animal Care and Use Committee of Northwestern Polytechnical University (Xi'an, China) and performed in accordance with the Institutional Ethical Guideline of Experimental Animals. Healthy 6 weeks old male *BALB/c* mice, weighting 22.85 ± 1.32 g, were obtained from the Experimental Animal Center of the Fourth Military Medical University (Xi'an, China) and housed individually in a polypropylene cage under standard laboratory conditions of 22 ± 1°C and a 12 h light-dark cycle (lights on from 06:00 a.m. to 18:00 p.m.) in the pathogen-free animal facility. All mice were *ad libitum* access to sterile water and commercial fodder free of probiotics and antibiotics (Keaoxieli, Beijing, China).

### Treatment of Animals With *Spirulina*

The fresh aqueous *spirulina* suspension was prepared daily at room temperature by adding 1.05 g or 2.10 g of dark blue-green spray-dried *Arthrospira platensis* (*A. platensis*) powder (Templer, Zhongshan, China) into 15 mL 0.9% sterile physiological saline (SCR, Shanghai, China). After 1 week of acclimatization, *BALB/c* mice were randomly assigned into one of the following treatment groups: (1) saline alone; (2) low dosage 1.5 g/kg of *spirulina*; and (3) high dosage 3.0 g/kg of *spirulina*. The saline alone or *spirulina* suspension was fed into the mice stomach with an oral gavage needle (12 Ga ×55 mm, 1.2 mm tip) (Hengao, Beijing, China) once daily for 24 consecutive days. The overall health of all treated mice was monitored closely, and their body weights were recorded every 3 or 4 days.

### Collection of Serum, Feces, and Cecum Samples

Before and during the treatment, every animal was raised separately in a metabolic cage (Suhang, Suzhou, China) and their fresh stools were collected at the following designated days: 0, 7, 14, and 21 days and the cecal contents were obtained at the end-point of treatments on the 25th day. The animals were deprived of food for 12 h and then anesthetized with 1.9% diethyl ether. The whole blood samples were withdrawn by *inferior vena cava* puncture and collected in polypropylene tubes (Shenggong, Shanghai, China). The serum was obtained by allowing the whole blood to clot and then centrifuging samples at 1,000 × *g* for 10 min in a refrigerated centrifuge (Thermo Scientific, USA). The resulting supernatant was immediately transferred into a clean polypropylene tube (Sangon Biotech®, Shanghai, China) and stored at −20°C for biomarker analysis. The cecum was excised, and its contents were collected in freezing tubes (IMEC Sunshinebio, Hangzhou, China). All fecal and cecum samples were snap frozen with liquid nitrogen and stored at −80°C for microbiota analysis.

### DNA Extraction, PCR Amplification, and Pyrosequencing

Total bacterial genomic DNA from the frozen fecal pellets and cecal specimens were extracted using QIAamp Fast DNA Stool MiniKit (QIAGEN, Germany). The microbial 16S rDNA was amplified with specific primers (*341F: ACT CCT ACG GGRSGC AGC AG, 806R: GGA CTA CVV GGG TAT CTA ATC*) targeting the V3-4 region by KAPA HiFi Hotstart Ready Mix PCR kit (Kapabiosystems, USA), purified with the AxyPrep DNA Gel Extraction Kit (Axygen, USA) and quantified by NanoDrop 2000 (Thermo Scientific, USA) at wavelengths of 260 and 280 nm. The library was finally fragment-selected and purified by 2% agarose gel electrophoresis. The purified fragments were end-repaired and ligated to the Illumina paired-end sequencing adapters. Amplicon libraries were sequenced on Illumina Miseq PE250 platform (Illumina, USA) for paired-end reads of 250 bp according to the Illumina instructions (Realbio Genomics Institute, Shanghai, China). The original data of high-throughput sequencing was taken to qualify preliminary screening using QIIME software (http://qiime.org/). The assembled long tags using the paired-end reads were quality controlled by removing tags with a length of <220 nt, an average quality score of <20 (low-quality bases), and tags containing >3 ambiguous bases by PANDAse (Li et al., 2016). A total of 3,132,725 clean reads were obtained after quality control. The deep sequencing data are available from the NCBI Sequence Read Archive under accession number PRJNA511783.

### Bioinformatics

A total of 51 fecal samples were collected on day of 7th, 14th, and 21st (number of samples and their ID: 5 S-7F, 6 L-7F, 6 H-7F; 5 S-14F, 6 L-14F, 6 H-14F; 5 S-21F, 6 L-21F, 6 H-21F) and 17 cecum samples were collected at the endpoint of the 25th day (number of samples and their ID: 5 S-25C, 6 L-25C, 6 H-25C). The singletons and chimeras from unique sequences were removed by UPARSE algorithm method (Edgar, 2013). After discarding the sequencing and amplification artifacts, the high-quality tags were clustered into operational taxonomic units (OTUs) with a similarity threshold of 97% using Usearch. The OTUs were further subjected to the taxonomy-based analysis by Ribosomal Database Project (RDP) algorithm. A total of 2,853,914 mapped reads were assigned to 416 OTUs, resulting in the classification of 86 taxa at the genus level. Each sample has 244 OTUs and 41,969 reads on average (Table S1 and Figure S1). α-diversity (Chao1, observed species, Shannon and Simpson diversity indexes) and β-diversity [weighted UniFrac, principal coordinate analysis (PCoA)] were analyzed using QIIME1 software. Linear discriminant analysis (LDA) effect size (LEfSe) method was performed with the LEfSe tool (http://huttenhower.sph.harvard.edu/galaxy).

### Evaluation of Blood Biomarkers

Serum levels of MDA, SOD, TC, and TG were quantified by biochemical assays of thiobarbituric acid (TBA), water-soluble tetrazolium-1 (WST-1), cholesterol oxidase p-aminophenol (COD-PAP), and glycerol-phosphate oxidase (GPO-PAP), respectively (Jiancheng Bioengineering Institute, Nanjing, China). Serum leptin was measured with Mouse Leptin ELISA Assay Kit (Jiancheng Bioengineering Institute, Nanjing, China). The procedures of each assay are performed according to the manufacturers' instructions (Jiancheng Bioengineering Institute, Nanjing, China). In brief, the oxidative stress was assessed in 20 μL serum by quantifying 1% TBA reactivity with MDA and WST-1 formazan for SOD enzyme activity. The resulting chromogen absorbance was determined at 532 nm for MDA-TBA adducts and at 435 nm for WST-1 formazan, respectively. The lipid contents (TC and TG) were measured directly in 0.25 mL serum by quantifying the formation of red quinone compound from COD-PAP and GPO-PAP at 510 nm. The quantitative measurement of serum leptin was determined by adding 50μL serum into 96-microplate wells containing the antibody complex of pre-coated anti-tag antibody, biotinylated anti-mouse leptin antibody, and the color density of horseradish peroxidase (HRP)-conjugated streptavidin was measured at 450 nm.

### Statistical Analyses

Differential analysis of various α-diversity indexes among samples within the same designated day were analyzed by the Wilcoxon signed-rank test (GraphPad Prism®, USA). Differences in the colonic bacterial structure, diversity and relative abundance among individual samples and treatment groups were analyzed by the multiresponse permutation procedure (MRPP) and Kruskal–Wallis tests, respectively, with *p* < 0.05 considered statistically significant. LEfSe method was used to identify differential bacterial taxa representing between groups at the genus level. The body weight and various blood biomarker levels were presented as mean ± standard deviation (SD) and were compared by *One-way factor analysis of variance (ANOVA)* following the Tukey *post-hoc method* with *p* < 0.05 considered statistically significant (GraphPad Prism®, USA). Spearman correlations between the differentially abundant microbiota and biological markers were computed in R, with the absolute value *r* > 0.7 and *p* < 0.033 considered to be statistically correlated with each other.

## Results

### *Spirulina* Affected the Diversity of Colonic Microbiota

**Figure 2:**
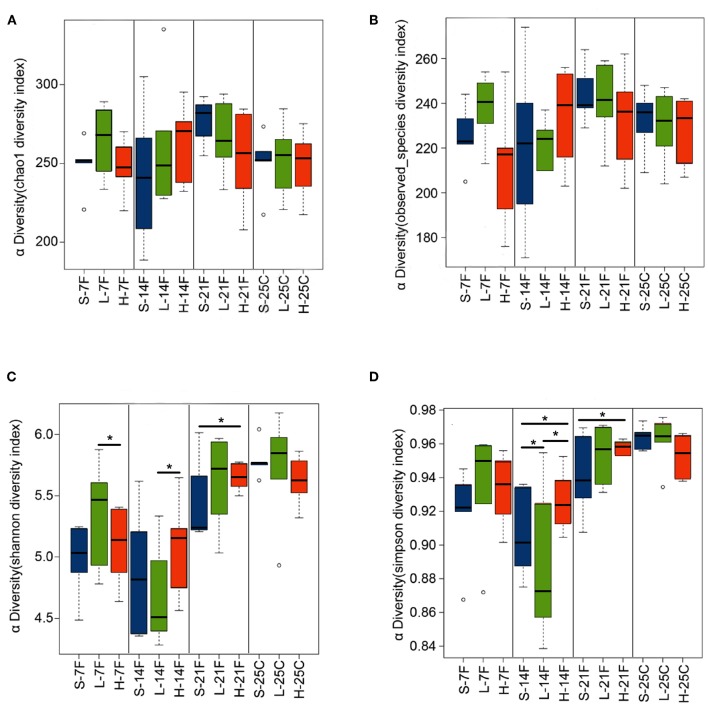
Barplots of α-diversity indexes in the treatment groups of saline, low, and high doses of *spirulina* at designated time points (day 7th, 14th, 21st, and 25th). **(A)** Chao1 index; **(B)** Observed species index; **(C)** Shannon index; **(D)** Simpson index. The difference of each α-diversity index was compared between individual treated samples within the same designated day by the Wilcoxon test with *p < 0.05 considered as statistically significant. In the x-axis label, S, L, and H represent the treatment of saline, low, and high doses of *spirulina*, respectively, and F and C represent feces and cecum, respectively.

**Figure 3:**
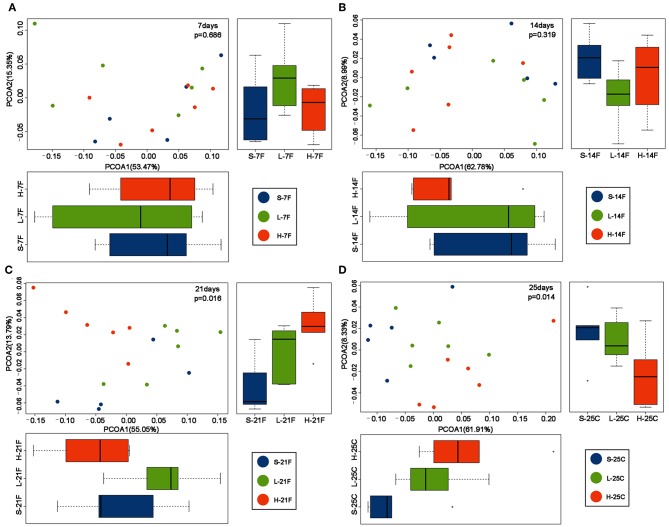
PCoA plots of β-diversity of feces collected on day **(A)** 7th day; **(B)** 14th day; **(C)** 21st; and cecal contents collected on day **(D)** 25th. Results revealed samples from *spirulina* treatment clustered separately from the saline group on the 21st and 25th days. The colonic microbial community structures were compared between treatments using the PCoA based on the weighted UniFrac distance matrixes. MRPP analysis was used to determine the statistical significance with *p* < 0.05. Each time point contains 5 or 6 samples. S (purple), L (green), and H (orange) represent saline, low, and high doses of *spirulina*, respectively, and F and C represent feces and cecum, respectively. Colored dots indicate the sample from each animal.

To assess the diversity and structural differences in the colonic microbiota of healthy mice treated with saline or *spirulina* at the low and high doses over 25 days of daily intervention, differential significance in α-and β-diversity of collected fecal and cecal samples were analyzed by the Wilcoxon signed-rank test and weighted UniFrac distance matrix based MRPP, respectively (Figures 2, 3). We firstly compared each of the α-diversity indexes (Chao1, Observed species, Shannon and Simpson) between treated samples (saline, low, and high doses of *spirulina*) on the same designated days (Figure 2). No significant difference in Chao1 (estimated the number of OTU) and observed species (observed the number of OTU) indexes were observed between samples from the treatments of saline, low, and high *spirulina* at all designated time points (Figures 2A,B), indicating the microbial richness (i.e., the total number of species) within each treatment were not changed. The Shannon and Simpson indexes, which took into account both richness and evenness (i.e., microbial equality) of species within each sample, showed different α-diversity on the 7th, 14th, and 21st days upon various *spirulina* doses (0 mg/kg, 1.5 mg/kg, 3 mg/kg) (Figures 2C,D). Especially, the Simpson index that weighted more on the dominant species revealed that intake of the low or high doses of *spirulina* exhibited different alteration patterns of the α-diversity (Figure 2D). Interestingly, the low dose of *spirulina* reduced the α-diversity on the 14th day and increased back on the 21st day, while the high-dose group exhibited an increase in the diversity over the time frame of 25 days (Figure S2). All of α-diversity indexes were not significantly different from each other in cecal samples on the 25th day (Figure 2). The structural difference in the colonic microbiota community was also observed by the gradual shifts of distance matrix among treatment groups over time using PCoA (Figure 3). At the early 7 and 14th days of *spirulina* intervention, no significant differences in colonic microbiota structure were observed with *p* = 0.686 and *p* = 0.319 (Figures 3A,B). At the later 21st and 25th days, the microbiota structure in fecal and cecal samples was significantly altered by *spirulina* treatments at different doses (both low and high doses) compared to the saline group with *p* = 0.016 and *p* = 0.014, respectively (Figures 3C,D), as demonstrated by the distinguished distances between treatment groups.

### *Spirulina* Modulated the Taxonomic Abundance of Colonic Microbiota

**Figure 4:**
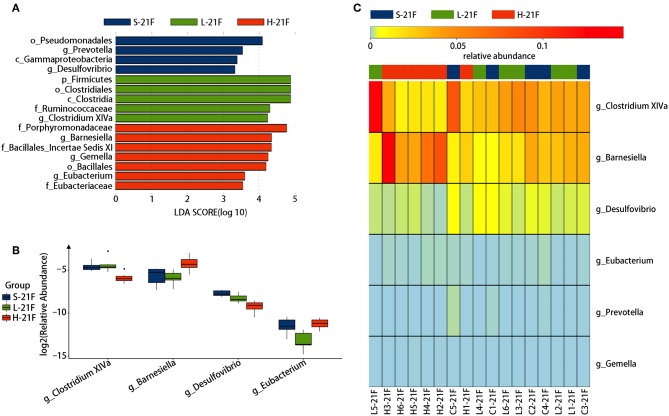
Significant influence of identified fecal microbiota at the genus level on the 21st day post-treatment of *spirulina* at low and high doses. **(A)** Histogram of the LDA scores of differential phylotypes abundant among treatment groups according to LEfse analysis; **(B)** Heatmap and **(C)** Barplots of the relative abundance of significantly differentiated bacteria among all three treatment groups. S-21F, L-21F, and H-21F represent fecal samples from saline, low, and high dose groups on day 21st, respectively. Differential significant analysis of abundance and composition of gut microbiome was analyzed at the OTUs level by the Kruskal-Wallis test.

**Figure 5:**
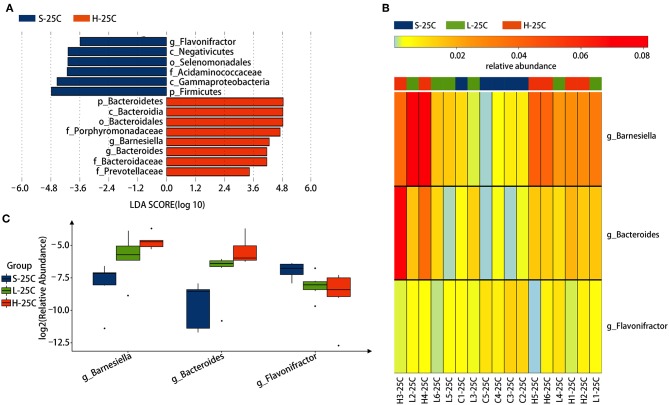
Significant influence of identified cecal microbiota at the genus level on the 25th day post-treatment of *spirulina* at low and high doses. **(A)** Histogram of the LDA scores of differential phylotypes abundant among treatment groups according to LEfse analysis; **(B)** Heatmap and **(C)** Barplots of the relative abundance of significantly differentiated bacteria among all three treatment groups. S-21C, L-21C, and H-21C represent cecal samples from saline, low, and high dose groups on day 25th, respectively. Differential significant analysis of abundance and composition of gut microbiome was analyzed at the OTUs level by the Kruskal-Wallis test.

To identify specific bacterial genus in *spirulina* treated groups at the low and high dose, the abundance of colonic bacteria from fecal pellets on the 21st day and cecal contents on the 25th day in mice were analyzed by the LEfSe method and Kruskal-Wallis test, and the significantly differentiated phylotypes by abundance was visualized using the histogram of LDA scores, heatmap and boxplots (Figures 4, 5). At the genus level of fecal bacteria, *Clostridium XIVa, Barnesiella, Desulfovribrio*, and *Eubacterium* were significantly differentiated by abundance among treatment groups (Figure 4A). The *Clostridium XIVa* genus in particularly was detected by LEfSe with a high LDA score (nearly four orders of magnitude), reflecting marked abundance in the low dose *spirulina* treatment group, whereas the *Barnesiella* and *Eubacterium* genera were found in abundance in the high-dose treated group (Figure 4A). Compared to saline-treated mice, the obvious changes in the colonic microbial composition and abundance was reduced *Eubacterium* and increased *Barnesiella* in the low and high doses treated *spirulina* groups, respectively (Figures 4B,C). At the genus level of cecal bacteria, only feeding high dose *spirulina* to mice significantly altered the gut microbial abundance and composition with increased the levels of *Barnesiella* and *Bacteroides* and decreased the level of *Flavonifractor* (Figure 5). These three bacteria taxa were consistently detected when comparing the saline group with the combined sample sets of the low- and high- doses *spirulina* (Figure S3).

### Intake of *Spirulina* Reduced the Oxidative Stress and Lipid Accumulation

**Figure 6:**
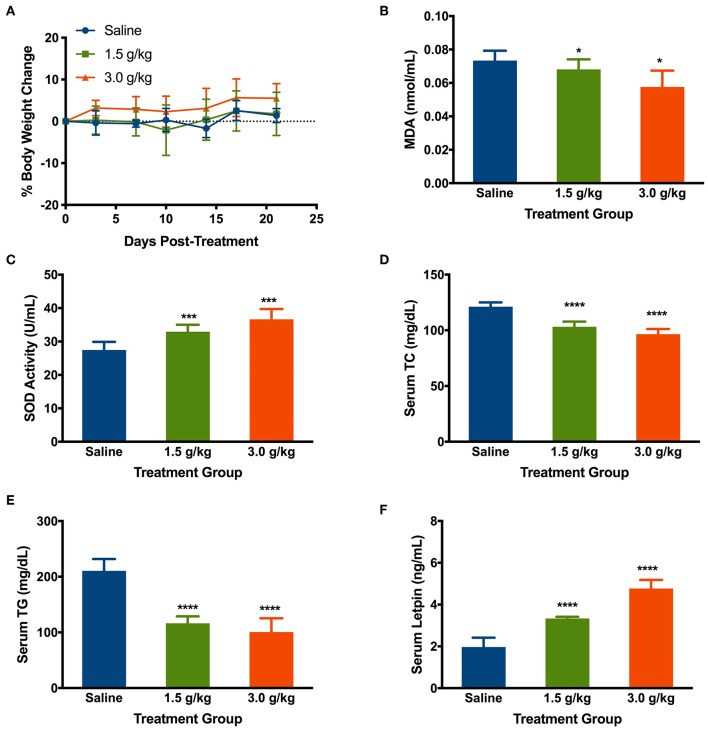
Effects of orally administering *spirulina* at low (1.5 g/kg) and high (3.0 g/kg) doses on various health factors in healthy mice. **(A)** The percent of body weight change in mice over 25 days post-treatments. The change of body weight in each mouse was determined by comparing them to the initial weight of the mice before the treatment; **(B)** Serum level of Malondialdehyde (MDA); **(C)** Serum level of superoxide dismutase (SOD); **(D)** Serum level of total cholesterol (TC); **(E)** Serum level of total triglycerides (TG); and **(F)** Serum level of Leptin. All mice were gavaged and monitored for 25 consecutive days post-treatment. At the end point of mice, the serum was obtained on the 25th day post-treatment of *spirulina*. Data are represented as mean ± SD with *n* = 7 *per* treatment group, and were analyzed by *one-way ANOVA* followed by the Tukey *post-hoc* method with *p < 0.05, ***p < 0.001, and ****p < 0.0001.

The change in body weight of mice treated with the saline, low, and high doses of *spirulina* at 1.5 g/kg and 3 g/kg, respectively, were determined by comparing them to the initial body weight of the mice prior to the treatment on the day 0. Intake of saline and *spirulina* showed no significant change in the body weight in healthy mice with *p* > 0.05. Compared to the saline group, the mice treated with *spirulina* at both low and high doses showed a similar trend of the body weight change (Figure 6A). The effects of orally administered *spirulina* on the oxidative stress and fat accumulation were biochemically evaluated by the serum biomarkers. Compared to the saline treatment, mice treated with *spirulina* showed a significant decline in the level of lipid peroxidation quantified by its end-product MDA formation and the elevation of serum antioxidant enzyme SOD activity in the dose-dependent manner (Figures 6B,C). Moreover, the serum lipid profile, including the level of TC and TG, were significantly attenuated in *spirulina* treatment groups compared to the one in saline with a nearly 2.1-fold reduction in TG level observed in the high dose (3.0 g/kg) treatment of *spirulina* (Figures 6D,E). Interestingly, the serum leptin, an appetite-regulating hormone secreted by adipocytes for hunger inhibition, was doubled in concentration along with the dose increment of *spirulina* from 1.5 to 3 g/kg (Figure 6F).

### Differentially Abundant Cecal Bacteria in High-Dose *Spirulina* Treatment Were Correlated With the Health Indices

**Figure 7:**
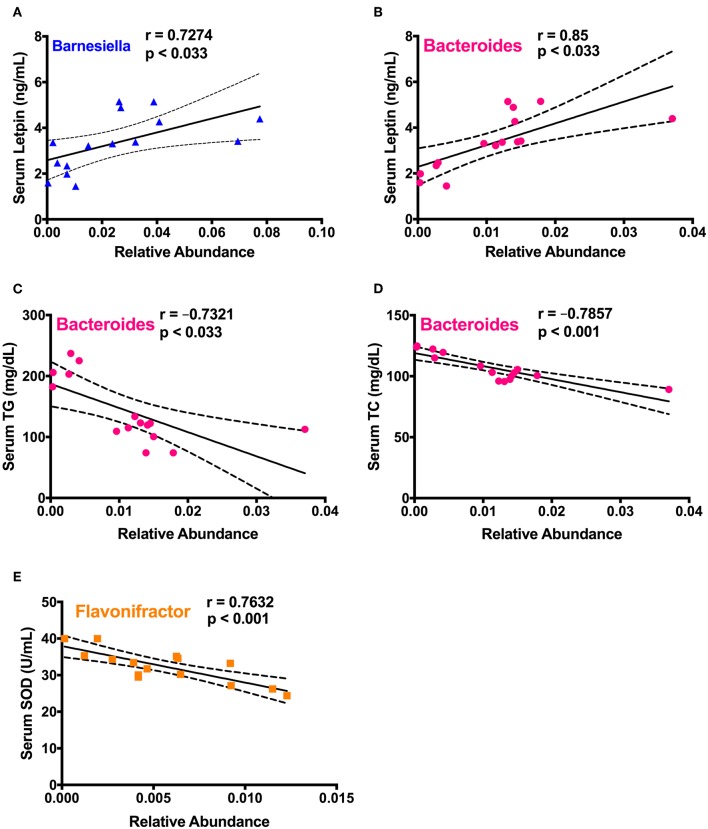
Spearman correlation analysis between the differentially abundant cecal microbial taxa and biological markers in mice. **(A)** *Barnesiella* and serum leptin level; **(B–D)** *Bacteroides* and the levels of serum leptin, TC, and TG, respectively; **(E)** *Flavonifractor* and serum SOD activity. Spearman correlation coefficient (r) and *p*-value were computed from a linear regression analysis in R, and a regression line with the 95% confidence interval were plotted. The absolute value of *r* > 0.7 and *p* < 0.033 were considered to be statistically correlated with each other.

To study the correlation between detected significantly different gut microbiota in cecum and prominent changes of various health biomarkers upon *spirulina* treatment on the 25th days, a series of spearman correlations were performed using a linear regression model (Figure 7 and Table S2). The relative abundances of *Barnesiella* and *Bacteroides* were positively correlated with the increased serum leptin level (Figures 7A,B). Also, the relative abundance of *Bacteroides* were negatively correlated with the serum lipid concentrations of TC and TG (Figures 7C,D). Moreover, the third identified bacteria, *Flavonifractor*, was negatively correlated with the activity of SOD, an important antioxidative enzyme providing cellular defense against reactive oxygen species (i.e., ${\text{O}}_{2}^{-}$) (Figure 7E).

## Discussion

The human gut microbiota plays a fundamental role in the well-being of their host, and is generally stable within individuals over time (Clemente et al., 2012). Yet, the intervention of the diet, food supplements and drugs (e.g., antibiotics) can perturb the dynamic of gut microbiota, resulting in a temporal shift of biological responses and sometimes the development of diseases in the host for a long term usage (Jernberg et al., 2007; Wu et al., 2011; Clemente et al., 2012; Pakpour et al., 2017; Quigley, 2018). Particularly, the influence of widely consumed yet not completely characterized nutraceuticals on the gut microbiota and their underlying mechanisms of biological functions remain largely unknown. In the present study, the dose effects of oral administered *spirulina* on the colonic microbiota community and physiological responses were observed in healthy mice (Figure 1). The microbial (evenness, structure and relative abundance) were significantly altered in groups between various doses of *spirulina* (Figures 2–5), assuming no difference in microbiome samples in all mice before *spirulina* treatment (i.e., on the day 0). The mice treated with low dose *spirulina* showed a short period of significantly decreased microbial α-diversity at the day 14th which were not observed in the high-dose treated group (Figure S2). Such varied patterns of temporal shift in α-diversity between low and high doses could result from intra- and inter-individual heterogeneity of the gut flora in mice even prior to any *spirulina* treatment (Laukens et al., 2016; Franklin and Ericsson, 2017). Yet, the oral administered low dose of *spirulina* (i.e., 1.5 g/kg) seem to increase the ratio of *Firmicutes* and *Bacteroidetes* from fecal pellets on the 21st day in contrast to the ones treated with the high dose (i.e., 3.0 g/kg) as evidenced by the change in the relative abundance of the two phyla (Figure S4). Such ratio change (*Firmicutes*/*Bacteroidetes*) has been shown to associate with obesity, IBD and colorectal cancer and gut physiological barrier structure (Ley et al., 2005; Islam et al., 2011; Devkota et al., 2012; Feng et al., 2015; Hayes et al., 2018). Thus, depending on the therapeutic application, rationally adjusting the dose of *spirulina* suspension is necessary.

Restoring a specific single bacterial species has been shown to improve therapeutic effects or reduce the disease symptoms (Geller et al., 2017; Roy and Trinchieri, 2017; Tsoi et al., 2017). The examples include the boost of intestinal colonization of *Barnesiella* for reduction of vancomycin-resistant enterococcus (Ubeda et al., 2013; Crouzet et al., 2015), increased mutualism *Bacteroides* for the human fitness and carbohydrate fermentation for other intestinal bacteria (Wexler, 2007; Wexler and Goodman, 2017), association of increased *Flavonifractor* with mental disease bipolar disorder and induction of oxidative stress and inflammation (Coello et al., 2019). However, unlike the definitive prebiotics (e.g., inulin, fructo-oligosaccharides, and galacto-oligosaccharides) for which their structural biochemistry and selectivity of specific bacterial strain, such as *Lactobacillus* and *Biofidobacterium*, are well-characterized for conferring health benefits (Gibson et al., 2017; Quigley, 2018), *spirulina* is a complex organism enriched of potential prebiotics, including carbohydrates, polyphenols, and polyunsaturated fatty acids (Tokuşoglu and Üunal, 2003; Khan et al., 2005; de Jesus Raposo et al., 2016). Knowing the selectivity of *spirulina* components to particular bacterial taxa may provide insights into the development of novel targeted nutraceutical-based drug delivery systems (Zhang et al., 2017a,b, 2018; Wang et al., 2018). Moreover, to maintain or improve the health benefits of the host, it is important to rationally dose *spirulina* for the *overall* homeostasis and holistic interaction of gut microbiota community rather than modulation of single bacterial species.

The changed levels of metabolic parameters (MDA, SOD, TG, TC, and leptin) in the *spirulina* treated mice indicate potential therapeutic application of *spirulina*-based therapies in the prevention and treatment of cancer, cardiovascular diseases, and obesity (Figures 6, 7), which are similar to previous reported antioxidative, anti-obesity, and anti-inflammatory effects of *spirulina* treatment *in vivo* (Piñero Estrada et al., 2001; Sharma et al., 2007; Yogianti et al., 2014; Yusuf et al., 2016; Neyrinck et al., 2017; Abd El-Hakim et al., 2018; Heo and Choung, 2018; Kata et al., 2018). More importantly, three differentially abundant cecal bacterial genus (*Barnesiella, Bacteroides*, and *Flavonifractor*) in the high dose treatment, not the low-dose, were significantly correlated with several health markers. Both *Barnesiella* and *Bacterioides* play an important role in the carbohydrate metabolism linking to the obesity (Hooper et al., 2002; Ley et al., 2005; Turnbaugh et al., 2006). The ability of boosting the leptin level and reducing the lipid metabolism by the high-dose *spirulina* implicates the potential therapeutic application for clinical obesity management (Carlier et al., 2010; Brown et al., 2014; Coello et al., 2019). Yet, the cause-and-effect relationship between the use of *spirulina* and its components (e.g., polysaccharides), the gut microbiota composition, and the physiological status of the host remains unclear. To assess the microbial functions, in-depth future investigation that utilize metabolomics, metagenomics, and transcriptomics is needed. Other factors such as the dosage regimen (short vs. long duration), sex (male vs. female), and biogeography of gut microbiota (distal vs. proximal colon) also need to be carefully considered in evaluating the therapeutic outcomes of *spirulina*.

In conclusion, daily orally administering *spirulina* provided the dose-dependent effects on colonic microbiota community in healthy mice and significantly changed the physiological states of oxidative stress, lipid profiles, and the appetite controlling hormone leptin. These findings shed light on *spirulina* induced biological functions potentially mediated through the gut microbiota, which in turn may lead to the novel and effective *spirulina* based pharmaceutical formulation to selectively modulate the gut microbial community in a controllable and precise manner for disease prevention and treatment.

## Ethics Statement

This study was carried out in accordance with the recommendations of the Institutional Ethical Guideline of Experimental Animals, the Institutional Animal Care and Use Committee of Northwestern Polytechnical University (Xi'an, China). The protocol was approved by the Institutional Animal Care and Use Committee of Northwestern Polytechnical University (Xi'an, China).

## Author Contributions

JH conducted experiments and wrote the first draft of manuscript. YL, ZP, and JL collected and analyzed the data. SP, SW, and QW contributed to analysis of 16S rDNA sequencing of gut microbiota. JS and HC reviewed the manuscript. RXZ proposed the initial idea, designed and supervised the studies, and wrote and proof the manuscript.

### Conflict of Interest Statement

The authors declare that the research was conducted in the absence of any commercial or financial relationships that could be construed as a potential conflict of interest.
