# Supplementary material for: Dose Effects of Orally Administered Spirulina Suspension on Colonic Microbiota in Healthy Mice
Source: Front Cell Infect Microbiol. 2019 Jul 5;9:243. doi: 10.3389/fcimb.2019.00243 (PMC6624478; doi:10.3389/fcimb.2019.00243)
Supplement: Supplementary file 1 [file Table_1.DOCX]

**Supporting Information**

**Dose Effects of Orally Administered *Spirulina* Suspension on Colonic Microbiota in Healthy Mice**

Jinlu Hu^1^, Yaguang Li^2^ Sepideh Pakpour^3^, Sufang Wang^1^, Zhenhong Pan^1,4^, Junhong Liu^1,4^, Qingxia Wei^2,5^, Junjun She^2^, Huaixing Cang^1*^, Rui Xue Zhang^1,2,4 *^

^1^School of Life Sciences, Northwestern Polytechnical University, Xi’an, Shaanxi, China.

^2^Department of General Surgery, The First Affiliated Hospital of Xi’an Jiaotong University, Xi’an, Shaanxi, China.

^3^Faculty of Applied Science, University of British Columbia, Kelowna, BC, Canada.

^4^Institute of Medical Research, Northwestern Polytechnical University, Xi’an, Shaanxi, China.

^5^Princess Margaret Cancer Center, University of Health Network, Toronto, ON, Canada.

^*^Correspondence:

Prof. Rui Xue Zhang

E-mail: [zhangruixue@nwpu.edu.cn](mailto:zhangruixue@nwpu.edu.cn)

Tel: +86-29-68750205

Prof. Huaixing Cang

E-mail: [hxcang@nwpu.edu.cn](mailto:hxcang@nwpu.edu.cn)

**Table S1. Operational taxonomic units (OTUs) of tested samples from each treatment group.**

| **Sample**  **Name** | **Sample Type** | **Group** | **Clean Reads** | **Mapped Reads** | **Mapped Ratio (%)** | **OTUs** |
| --- | --- | --- | --- | --- | --- | --- |
| S1-14F | fecal | saline | 41486 | 39455 | 95.10437 | 249 |
| S1-21F | fecal | saline | 46954 | 44468 | 94.70546 | 243 |
| S1-25C | cecum | saline | 33269 | 29896 | 89.86143 | 240 |
| S1-7F | fecal | saline | 49574 | 43974 | 88.70376 | 240 |
| S2-14F | fecal | saline | 39148 | 36334 | 92.81189 | 231 |
| S2-21F | fecal | saline | 45493 | 41862 | 92.01855 | 268 |
| S2-25C | cecum | saline | 52568 | 45553 | 86.65538 | 259 |
| S2-7F | fecal | saline | 44292 | 40211 | 90.78615 | 235 |
| S3-14F | fecal | saline | 48397 | 45428 | 93.86532 | 288 |
| S3-21F | fecal | saline | 58280 | 53814 | 92.33699 | 268 |
| S3-25C | cecum | saline | 53356 | 46573 | 87.28728 | 243 |
| S3-7F | fecal | saline | 47252 | 42314 | 89.54965 | 253 |
| S4-14F | fecal | saline | 53828 | 52199 | 96.97369 | 229 |
| S4-21F | fecal | saline | 54093 | 50362 | 93.10262 | 289 |
| S4-25C | cecum | saline | 38017 | 36519 | 96.05966 | 255 |
| S4-7F | fecal | saline | 44542 | 41480 | 93.12559 | 232 |
| S5-14F | fecal | saline | 36585 | 35203 | 96.2225 | 185 |
| S5-21F | fecal | saline | 52921 | 50705 | 95.81263 | 258 |
| S5-25C | cecum | saline | 49178 | 45171 | 91.85205 | 228 |
| S5-7F | fecal | saline | 46686 | 41058 | 87.94499 | 224 |
| H1-14F | fecal | high | 47376 | 46254 | 97.63171 | 263 |
| H1-21F | fecal | high | 45111 | 40153 | 89.00933 | 255 |
| H1-25C | cecum | high | 46703 | 41746 | 89.38612 | 250 |
| H1-7F | fecal | high | 57024 | 51478 | 90.27427 | 236 |
| H2-14F | fecal | high | 35517 | 31835 | 89.63313 | 255 |
| H2-21F | fecal | high | 43792 | 37418 | 85.44483 | 221 |
| H2-25C | cecum | high | 47293 | 43548 | 92.08128 | 250 |
| H2-7F | fecal | high | 46365 | 41077 | 88.59485 | 228 |
| H3-14F | fecal | high | 36939 | 35434 | 95.92572 | 230 |
| H3-21F | fecal | high | 51288 | 45346 | 88.41444 | 240 |
| H3-25C | cecum | high | 43714 | 40190 | 91.93851 | 225 |
| H3-7F | fecal | high | 50904 | 46585 | 91.5154 | 210 |
| H4-14F | fecal | high | 43853 | 41201 | 93.95252 | 214 |
| H4-21F | fecal | high | 40180 | 36549 | 90.96317 | 208 |
| H4-25C | cecum | high | 42768 | 40234 | 94.07501 | 240 |
| H4-7F | fecal | high | 45500 | 41886 | 92.05714 | 244 |
| H5-14F | fecal | high | 56480 | 52448 | 92.86119 | 292 |
| H5-21F | fecal | high | 33046 | 29889 | 90.44665 | 263 |
| H5-25C | cecum | high | 42596 | 39292 | 92.2434 | 227 |
| H5-7F | fecal | high | 42826 | 41258 | 96.33867 | 185 |
| H6-14F | fecal | high | 52683 | 49012 | 93.03191 | 256 |
| H6-21F | fecal | high | 56608 | 49422 | 87.30568 | 252 |
| H6-25C | cecum | high | 30777 | 27335 | 88.81632 | 241 |
| H6-7F | fecal | high | 45252 | 41921 | 92.639 | 270 |
| L1-14F | fecal | low | 42210 | 38304 | 90.74627 | 230 |
| L1-21F | fecal | low | 45337 | 40077 | 88.398 | 265 |
| L1-25C | cecum | low | 47486 | 41274 | 86.91825 | 261 |
| L1-7F | fecal | low | 52078 | 46410 | 89.11633 | 251 |
| L2-14F | fecal | low | 42834 | 40410 | 94.34094 | 246 |
| L2-21F | fecal | low | 38575 | 34598 | 89.69021 | 249 |
| L2-25C | cecum | low | 60211 | 53874 | 89.47535 | 265 |
| L2-7F | fecal | low | 46890 | 39672 | 84.60653 | 267 |
| L3-14F | fecal | low | 43845 | 41874 | 95.50462 | 244 |
| L3-21F | fecal | low | 55498 | 49175 | 88.6068 | 275 |
| L3-25C | cecum | low | 49598 | 44004 | 88.72132 | 234 |
| L3-7F | fecal | low | 50214 | 45396 | 90.40507 | 247 |
| L4-14F | fecal | low | 37549 | 36444 | 97.05718 | 250 |
| L4-21F | fecal | low | 51207 | 45473 | 88.80231 | 251 |
| L4-25C | cecum | low | 51294 | 43678 | 85.15226 | 256 |
| L4-7F | fecal | low | 45483 | 38654 | 84.9856 | 255 |
| L5-14F | fecal | low | 50753 | 45373 | 89.39964 | 226 |
| L5-21F | fecal | low | 48305 | 43429 | 89.90581 | 231 |
| L5-25C | cecum | low | 48878 | 46192 | 94.50469 | 219 |
| L5-7F | fecal | low | 33500 | 30784 | 91.89254 | 223 |
| L6-14F | fecal | low | 49394 | 46940 | 95.03179 | 226 |
| L6-21F | fecal | low | 47844 | 42546 | 88.92651 | 248 |
| L6-25C | cecum | low | 43037 | 38387 | 89.19534 | 227 |
| L6-7F | fecal | low | 30191 | 26856 | 88.95366 | 246 |


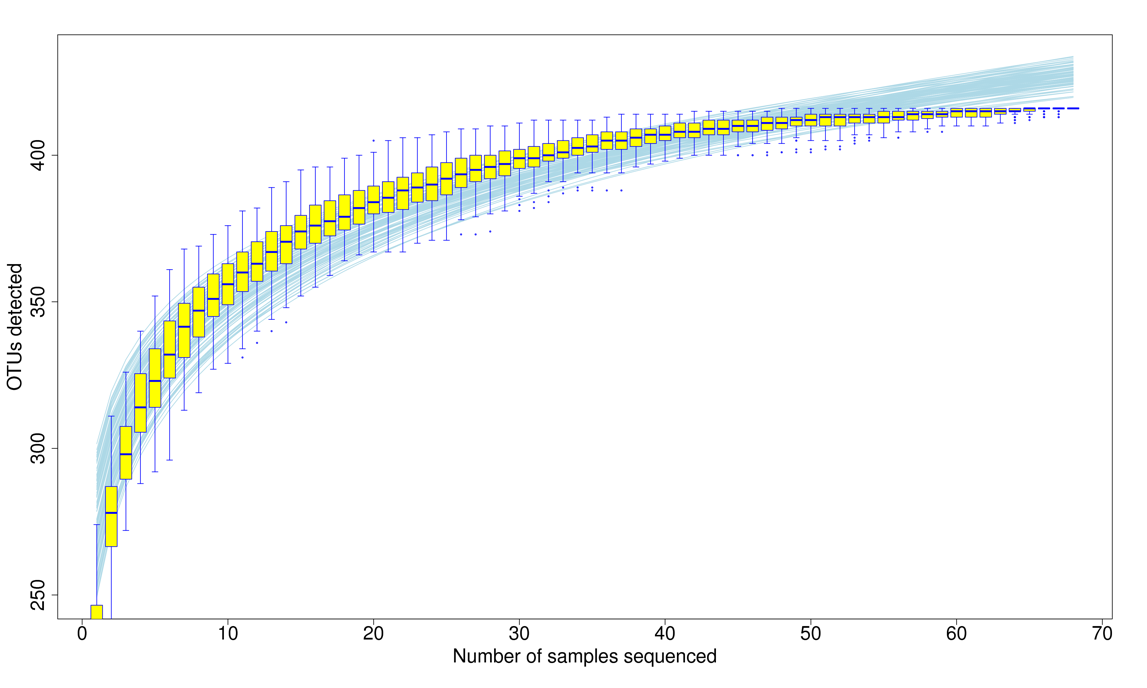


**Figure S1.** Individual rarefaction curves of fecal and cecal samples. The observed OTUs almost reached the plateau.


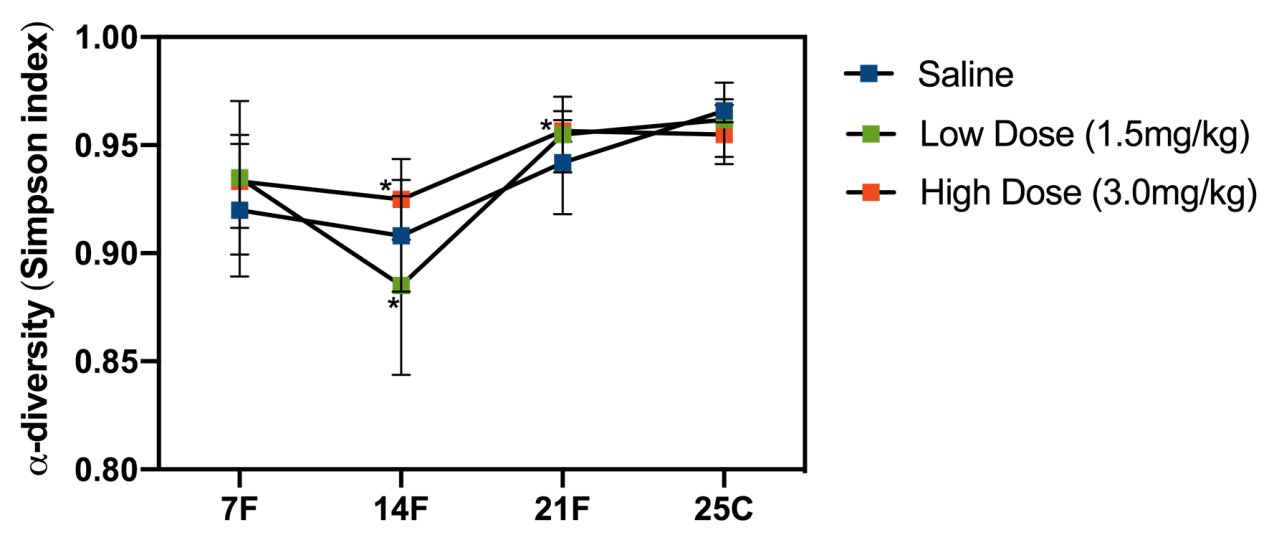


**Figure S2.** α-diversity (Simpson index) of the treatment groups (saline, low, and high doses of *spirulina*) at designated time points (day 7^th^, 14^th^, 21^st^ and 25^th^). The difference of each α-diversity index was compared between individual treated samples within the same designated day by the Wilcoxon test, with p-value <0.05 considered as statistically significant. F and C represent feces and cecum, respectively.


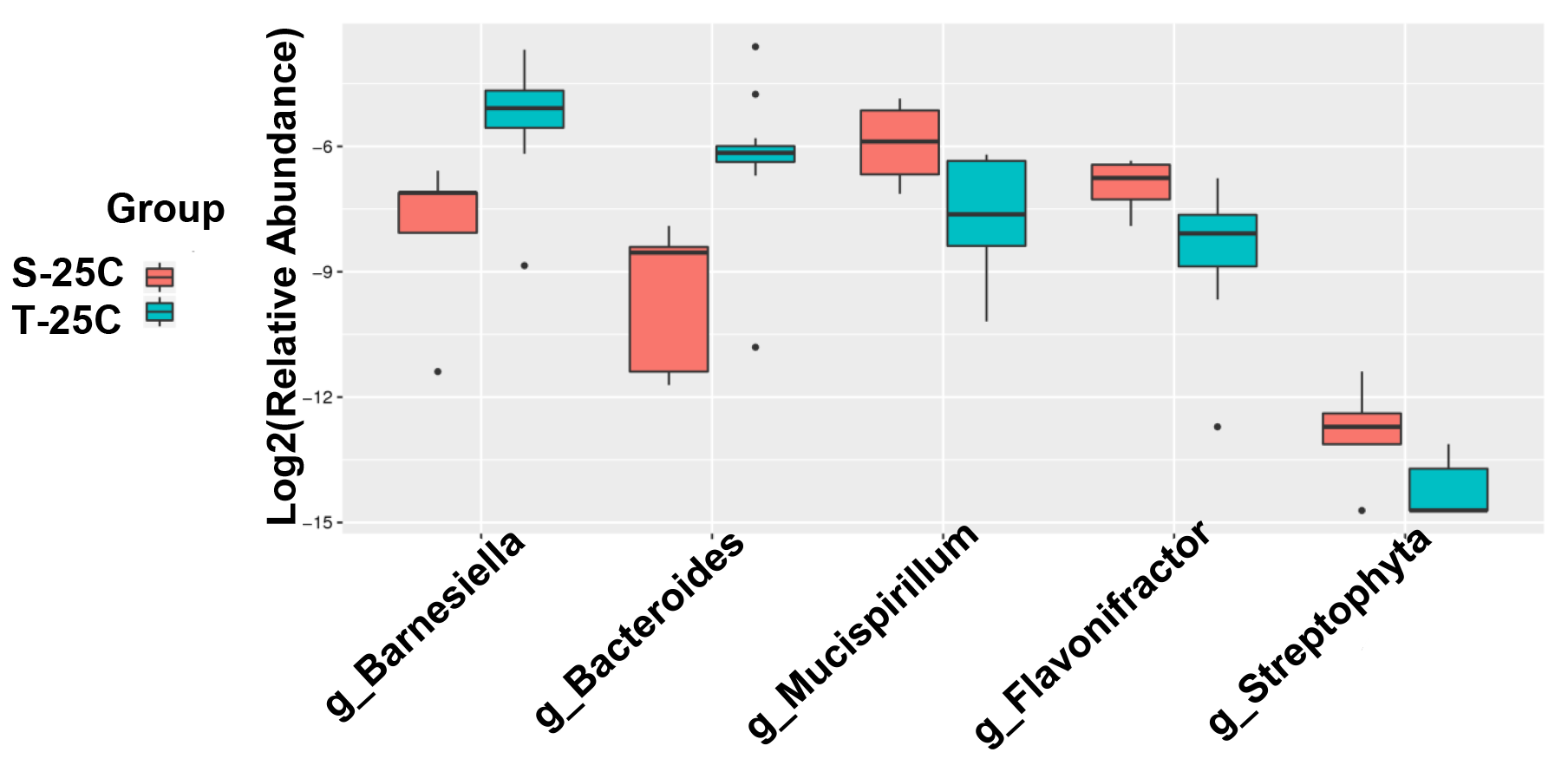


**Figure S3.** Barplots of the relative abundance of significantly differentiated bacteria from cecal contents. S (orange) and T (green) represent saline and *spirulina*-treatment (low and high doses combined), respectively. C represents feces and cecum, respectively. The cecal contents were collected from healthy male mice on the 25^th^ day post-treatment.


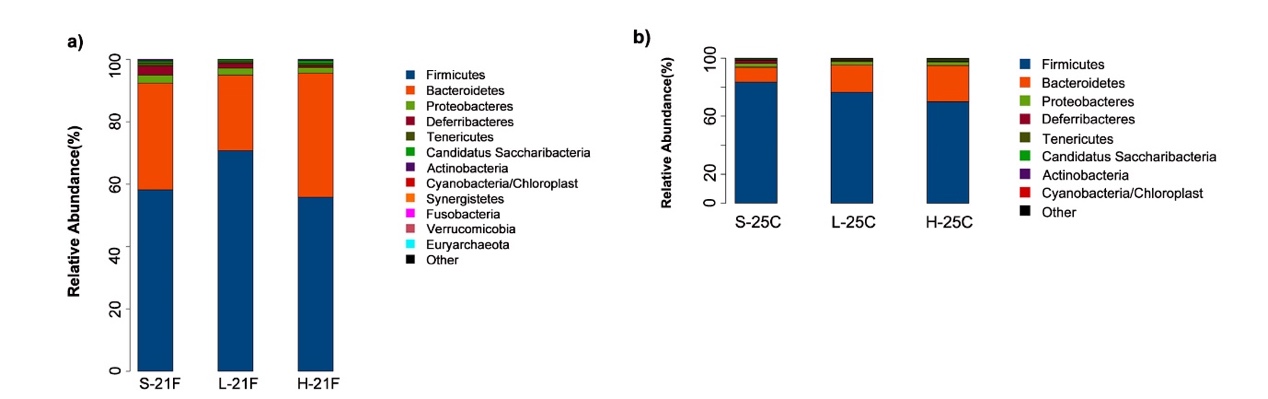


**Figure S4.** Phylum level abundance of gut bacteria from a) fecal pellets on the 21^st^ day and b) cecal contents on the 25^th^ day post-treatment. S, L, and H represent saline, low, and high doses of *spirulina* treatments, respectively. Among the bacterial groups, *Firmicutes* and *Bacteroidetes* were two dominant phyla contributing 58% and 34% in feces and 83% and 10% of cecum respectively in the saline treated group. At low dose treatment of *spirulina*, the relative abundance of *Firmicutes* and *Bacteroidetes* in feces were increased to 71% and decreased to 24%, respectively, whereas at high dose treatment, the relative abundance of *Firmicutes* and *Bacteroidetes* in cecum were decreased up to 70% and increased up to 25%, respectively.

**Table S2.** Spearman correlation coefficient (r) and p-value between differential abundant microbiota taxa and health related factors. The absolute value of r>0.7 and p-value <0.033 are required to be considered as statistically correlated with each other. ^*^ p-value <0.033, ^**^ p-value <0.002, and ^***^p-value <0.001.

|  | ***Barnesiella*** | ***Bacteroides*** | ***Flavonifractor*** |
| --- | --- | --- | --- |
| **MDA** | r = -0.3557  p=0.1956 | r= -0.5107  p=0.0099 (*) | r= 0.2698  p=0.55 |
| **SOD** | R = -0.4812  p=0.1567 | r= -0.6452  p= 0.0253 (*) | r= 0.7674  p= 0.0003 (***) |
| **TC** | r= -0.6148  p= 0.01138 (*) | r= -0.7857  p= 0.000046 (***) | r= 0.6416  p= 0.0051 (*) |
| **TG** | r= -0.6488  p= 0.02233 (*) | r= -0.7321  p= 0.0053 (*) | r= 0.3825  p=0.0615 |
| **Leptin** | r= 0.7274  p= 0.02283 (*) | r= 0.85  p= 0.0028 (*) | r= -0.4754  p=0.0678 |
